# Effects of the allopathic and osteopathic graduate medical education merger on U.S. specialty training: a review

**DOI:** 10.1080/10872981.2025.2605382

**Published:** 2026-01-30

**Authors:** Sarah Ibrahim, Mohamed Abdelhady, Jacqueline Venckus, Caleb North, Forrest Bohler

**Affiliations:** a Oakland University William Beaumont School of Medicine, Rochester, MI, USA; b Western University of Health Sciences College of Osteopathic Medicine of the Pacific, Pomona, CA, USA; c College of Letters and Science, Montana State University, Bozeman, Montana, USA

**Keywords:** Graduate medical education, ACGME, American Osteopathic Association, Single Accreditation System Merger, residency

## Abstract

The Single Accreditation System (SAS) unified graduate medical education (GME) accreditation for allopathic (MD) and osteopathic (DO) programs under the Accreditation Council for Graduate Medical Education (ACGME). Implemented between 2015 and 2020, it aimed to expand access and standardize residency training across degree types. While the SAS succeeded in expanding opportunities for DO graduates in certain specialties, particularly family medicine and pathology, persistent disparities remain across competitive medical and surgical fields. Specialty-specific analyses reveal that DO applicants continue to face significant barriers in dermatology, ophthalmology, plastic surgery, neurosurgery, and orthopedic surgery, with disproportionately lower match rates and limited representation in top-tier residency programs. Structural challenges including the closure of many osteopathic-led programs, limited access to research mentorship, and degree-based bias among residency programs have exacerbated these disparities. Although Osteopathic Recognition and initiatives such as the Pathologist Pipeline have helped support osteopathic participation in select areas, broader reforms are needed to fulfill the original goals of the SAS. Enhancing academic partnerships, expanding research infrastructure, addressing implicit biases, and fostering DO leadership within academic medicine are critical steps toward ensuring equitable residency access for all graduates. Continued monitoring of match trends and specialty-specific outcomes will be essential to assessing the SAS’s long-term impact on healthcare workforce diversity and equity.

## Introduction

Graduate medical education (GME) in the U.S. historically operated under two distinct accreditation systems: one overseen by the Accreditation Council for Graduate Medical Education (ACGME) for allopathic (MD) programmes and another by the American Osteopathic Association (AOA) for osteopathic (DO) programmes [1]. Traditionally, DO graduates were able to apply to both AOA-accredited residencies as well as ACGME-accredited programmes. MDs were limited to ACGME-accredited programmes and were barred from AOA programmes [2].

However, several converging pressures underscored the need for a unified accreditation framework. The rapid proliferation of osteopathic medical schools whose graduating class sizes outpaced available AOA-accredited GME positions, the ACGME’s 2013 subspecialty eligibility restrictions on AOA programmes, and declining interest in primary care among DO graduates all contributed to mounting strain on the system [2,3].

To address these challenges, the Single Accreditation System (SAS) was formally launched following a memorandum of understanding signed on August 26, 2014, by the AOA, the American Association of Colleges of Osteopathic Medicine (AACOM), and the ACGME, with implementation beginning July 1, 2015, and concluding on June 30, 2020 [4]. The SAS aimed to unify standards for residency training, reduce redundant accreditation efforts, minimise institutional costs, and ensure equitable application access for both MD and DO graduates [4]. Advocates, including the AOA, framed the initiative as a means to enhance training quality and expand DO access to residency positions [3,5].

The primary objective of this narrative review is to synthesise existing peer-reviewed literature on the effects of the SAS across selected specialties, with particular attention to trends in equity, access to training opportunities, and specialty-specific outcomes in the post-SAS era.

### Search strategy

To ensure a comprehensive and evidence-based review, we conducted a targeted literature search using PubMed database between January and April 2025. Search terms included combinations of ‘Single Accreditation System,’ ‘AOA and ACGME merger,’ ‘osteopathic match rates,’ ‘DO vs MD residency outcomes,’ and specialty-specific terms such as ‘dermatology,’ ‘plastic surgery,’ and ‘neurosurgery.’ Peer-reviewed articles, national match data reports from the National Resident Matching Programme (NRMP), publications from the AOA and ACGME, and official statistics from the Association of American Medical Colleges and AACOM were reviewed. Bibliographies of key articles were also manually screened for additional relevant sources. Data were prioritised based on recency, sample size, methodological quality, and relevance to specialty-specific trends in GME access and outcomes. Any medical or surgical specialty not discussed in this review were excluded due to the absence of primary, peer-reviewed research on the effects of the SAS identified during the literature search period.

**Figure 1.**
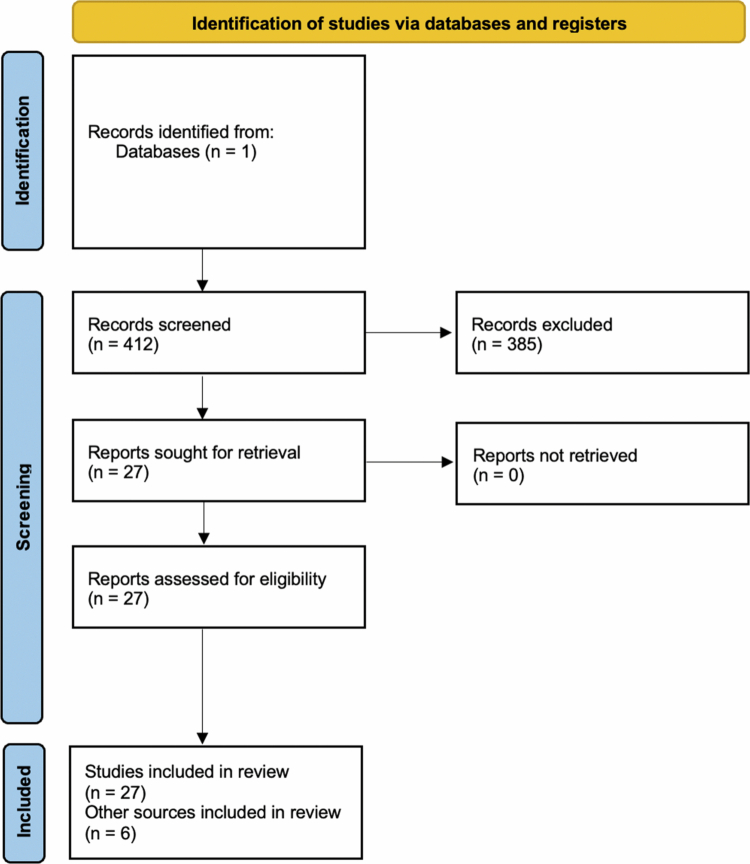
PRISMA-style flowchart of included studies.

The research question guiding this review was: ‘What has been the effect of the Single Accreditation System on residency access, match outcomes, and specialty-specific representation among DO and MD graduates across selected medical and surgical specialties?’ Inclusion criteria consisted of peer-reviewed studies or national data sources reporting outcomes related to residency match rates, programme representation, or specialty-specific effects following the implementation of the Single Accreditation System. Publications were excluded if they did not address residency outcomes or specialty distribution, lacked primary or specialty-specific data, or were outside the context of U.S. ACGME-accredited graduate medical education. The search period of January-April 2025 reflects the window during which data extraction and synthesis were performed; earlier studies published between 2015 and 2024 were included when relevant. A PRISMA-style flow diagram is included to visually summarise the search and screening process and illustrate the relevance-based inclusion and exclusion pathway used to identify studies for narrative synthesis (Figure 1).

### Family medicine

The implementation of the SAS significantly impacted osteopathic participation in family medicine (FM) residencies. During this time, the proportion of DOs matching into FM increased from 15% to 34%, reflecting the growing integration of osteopathic graduates into ACGME-accredited programmes (Table 1) [6]. Despite this progress, the transition presented notable challenges. Many smaller, rural AOA-accredited FM programmes struggled to meet the ACGME’s heightened accreditation standards, leading to programme closures that disproportionately affected medically underserved communities as osteopathic graduates have historically been more likely to practice in rural communities [6]. Additionally, training within allopathic environments has contributed to a decline in the clinical use of osteopathic manipulative treatment (OMT) among DOs [6].

**Table 1.** Summary of key findings.

Specialty	Key outcomes	MD vs DO comparison	Time period	source
Family Medicine	DO match proportion increased substantially	15% → 34% DOs in FM positions	2015–2020	Fugazzi & Cummings, 2022
Dermatology	DO match rates decreased	Down to 0.4%	2012–2019	Craig et al., 2021
Ophthalmology Programmes	Over half of osteopathic programmes lost	15 programmes → 7 programmes (53% attrition)	2015–2020	Ahmed et al., 2021
Ophthalmology Match Outcomes	DO representation declines	DO residents decreased post-merger	2016–2024	Bohler et al., 2024
Radiology	DO enrolment increasing	331 → 659 DO residents	2015–2023	NRMP/ACGME Reports
Pathology	DO representation doubled	6% → 12.1%	2011–2020	Jajosky et al., 2021
General Surgery	DO match rate decreased	59.4% → 53% DO	2020–2021	Williamson et al., 2024
Orthopaedic Surgery	DO match rate decreased	Down to 50.2%	2023	Modica et al., 2024
Plastic Surgery (Integrated)	Significant match disadvantage	0% DO vs 69.6% MD	2020	Etheart et al., 2021
Plastic Surgery Representation	Lower DO match rate	2.7% DO residents despite 25% of U.S. grads	2024	Raborn et al., 2024
Otolaryngology (ENT)	Lower DO match rate	51.5% DO vs 73.6% MD	2020	Etheart et al., 2021
Thoracic Surgery (Integrated)	Match disadvantage	12.5% DO vs 41% MD	2020	Etheart et al., 2021
Neurosurgery	Sharp decrease in DO residents post-SAS	14 DO → 4 DO	2016–2024	Ariwodo et al., 2024

To mitigate these losses and preserve osteopathic principles, the AOA, as part of its negotiations to join the SAS, secured the inclusion of ‘Osteopathic Recognition’ within the ACGME accreditation framework for programmes incorporating OMT and other tenets of osteopathic medicine. The same agreement stipulated that the ACGME formally recognise neuromusculoskeletal medicine as a distinct specialty with its own accreditation standards [6,7]. As of April 2025, 189 of the 814 ACGME-accredited family medicine programmes (23%) have attained ‘Osteopathic Recognition’ [8]. Evidence suggests that programme directors who implement formal OMT curricula rate their DO residents more favourably, emphasising the academic and clinical value of osteopathic training [6]. Recent growth of DOs within FM residencies suggest that DOs are in line to surpass MDs and international medical graduates (IMGs) in filling ACGME FM residency slots in the near future [9].

### Dermatology

Prior to the full implementation of the SAS from 2012-2016, DOs accounted for just 0.9% of residents in traditionally MD dermatology programmes [10]. While this figure rose to 4.4% between 2017 and 2019, much of the apparent progress was attributable to the reclassification of former AOA programmes under ACGME accreditation [10]. When isolating traditional MD programmes post-merger, DO match rates actually declined to 0.4% (Table 1) [10].

In 2020, DO applicants filled only 7.6% of all dermatology residency positions [11]. Dermatology remains one of the most competitive medical specialties, with programme directors prioritising not only board scores and letters of recommendation, but also demonstrated scholarly productivity, research involvement, and presentations at professional conferences. Given the organisational structure of most osteopathic medical schools, which often lack large, research-intensive academic medical centres, DO students may have fewer opportunities to build the type of research and academic portfolios that competitive dermatology programmes expect [12]. Now that all dermatology residencies are under the ACGME, DO applicants must meet the same high selection criteria as their MD peers, but without the benefit of institutional environments that typically facilitate these experiences, placing them at a relative disadvantage in the match process.

This research deficit is likely largely structural rather than individual. Most osteopathic schools lack home dermatology residency programmes or affiliated university hospitals with NIH-funded departments, making it difficult for students to access specialty-specific mentorship or participate in clinical trials. Additionally, osteopathic curricula emphasise community-based and primary care rotations, leaving limited elective time for research or national conference participation. These systemic limitations directly constrain scholarly output, which remains a heavily weighted selection metric in dermatology. To mitigate these barriers, reforms such as establishing formal research affiliations between osteopathic institutions and academic dermatology departments, incorporating protected research blocks within the clinical curriculum, and funding remote research collaborations could substantially improve DO applicants’ competitiveness in future cycles.

### Ophthalmology

Prior to the SAS merger, 15 ophthalmology residency programmes were accredited by the AOA [13]. However, only seven of these programmes successfully transitioned to ACGME accreditation, representing a 53% loss in osteopathic training programmes (Table 1) [13,14]. This reduction disproportionately affected rural areas, where osteopathic physicians are more likely to practice and where access to eye care services is already limited [13,15].

Multiple factors contributed to this attrition. 58% of the programmes cited inadequate hospital or administrative support, and 42% pointed to the prohibitive costs of meeting ACGME requirements as key barriers to transition [13]. The consolidation of programmes into urban academic centres further eroded opportunities for rural service and osteopathic participation. For example, 67% of DO ophthalmologists in Michigan were practicing in communities with populations under 50,000, compared to only 37% of MDs [16]. With fewer DO-led programmes surviving the transition physician shortages in underserved regions have likely been exacerbated. Further, findings show the number of DO residents in ophthalmology residency programmes have declined after the merger [14].

### Surgical specialties

For the purposes of this review, ‘surgical specialties’ refer to general surgery, orthopaedic surgery, neurosurgery, otolaryngology, plastic surgery, and cardiothoracic surgery. Unless otherwise noted, the data presented for surgical specialties do not encompass subspecialty fellowship positions. Any surgical subspecialty not discussed here, such as urology, is excluded due to the lack of primary research studies identified on the effects of the SAS at the time of this paper’s writing. Where year ranges appear discontinuous, this reflects limitations in the availability of primary specialty-specific data rather than inconsistencies in reporting; gaps between datasets correspond to periods for which no peer-reviewed outcomes were identified in the literature.

AOA surgical subspecialties also faced significant hurdles in the transition to ACGME accreditation resulting in closure of some programmes. Of the 159 AOA-accredited surgical residencies in 2015, 26% (41 programmes) were lost due to failure to apply or withdrawal from the accreditation process, with neurosurgery, ophthalmology, and otolaryngology among those experiencing the highest attrition rates [2,13,17]. Although the proportion of DOs in ACGME surgical residencies rose from 2.4% to 9.6% between 2015 and 2019, these gains were concentrated primarily in former AOA programmes [2].

With the discontinuation of the AOA match system in 2020, DO applicants applying solely through the NRMP saw a 3% decline in PGY-1 surgical specialty spots [18]. Within otolaryngology, only 51.5% of DO applicants matched compared to 73.6% of MD applicants (Table 1) [18]. In plastic surgery, match rates were 0% and 69.6% for DO and MD applicants, respectively [18]. In thoracic surgery, these rates were 12.5% for DOs versus 41% for MDs [18]. Even within general surgery, only 59.4% for DO applicants matched compared to 75% for MD applicants [18].

These challenges extend further in neurosurgery, where the number of DO residents has sharply declined from 14 in 2016 to just 4 in 2024 (Table 1) [17]. Only 16.7% of DO applicants matched compared to 74.4% of MD applicants [18]. A major factor contributing to this decline was the closure of AOA programmes as only four successfully transitioned to ACGME accreditation [17].

Furthermore, only 2.7% of plastic surgery residents are DOs despite DOs making up 25% of U.S. graduating medical students (Table 1) [19]. DO applicants were also more likely to match into lower-ranked plastics residency programmes compared to their MD counterparts as 10% of matched DO applicants secured a position at a top quartile programme versus 36.8% of MDs [19].

Prior to the full implementation of the ACGME merger, only 15 DO applicants matched into ACGME orthopaedic surgery residency positions (2%) [11]. After the merger, DO representation in orthopaedic surgery residencies grew to 13.2% in 2020 [20]. However, this increase largely reflects matches into former AOA programmes [20]. While the merger was intended to increase opportunities for both MD and DO graduates, more MDs have matched into previous AOA residency programmes than DOs have matched into traditional ACGME programmes. Since unification, 47.6% of former AOA orthopaedic surgery positions have been filled by MDs while DOs represent only 1.1% of residents in traditional allopathic programmes [10]. Moreover the match rate for DO students dropped to 50.2% in 2023 [21].

Within general surgery, the match rate for DO applicants decreased slightly from 2020 to 2021 from 59.4% to 53.0% (Table 1) [22]. In a 2021 survey of programme directors, 53% of programmes seldom or never interviewed a US DO applicant in 2021, a figure rising to 63% the following year [23]. Structural barriers, including biases against DO credentials, limited access to home residency programmes, and reduced away rotation opportunities during the COVID-19 pandemic, have likely disadvantaged DO applicants in these surgical specialties [24]. The COVID-19 pandemic likely amplified these disparities by eliminating many in-person sub-internships and limiting exposure opportunities at programmes unfamiliar with DO trainees. Several surgical specialties suspended away rotations entirely in 2020, forcing applicants to rely more heavily on home-institution advocacy, a factor that likely disproportionately disadvantaged students from osteopathic schools lacking affiliated surgical residencies [25,26].

### Radiology

Within radiology, concerns surrounding DO stigma remains. A 2017–2018 survey of 268 radiology residents found that DO respondents perceived their osteopathic degree as a disadvantage with many reporting they were actively discouraged from pursuing the specialty [27]. Furthermore, 90% of both MD and DO participants believed that residency selection processes favoured allopathic degrees [27]. DO residents also reported that their clinical abilities were more frequently questioned compared to their MD peers [27].

Prior to the SAS, most DOs in radiology were already training in ACGME-accredited programmes, though the AOA maintained a small number of its own residencies. In 2014–2015, the AOA had 16 accredited radiology programmes; 10 applied for ACGME accreditation, 2 later withdrew from the SAS process, and 8 ultimately achieved accreditation [28]. The number of DOs in ACGME radiology residencies was 331 in 2015–2016. Since the completion of the SAS, DO representation in radiology has steadily increased, with ACGME data showing 584 DO residents in 2020–2021, 635 in 2021–2022, and 659 in 2022–2023 (Table 1) [11]. This upward trend suggests that the SAS may have expanded access for osteopathic graduates in radiology, particularly as declining interest among US MD applicants has created additional openings. While survey data from 2017–2018 indicated that some DO residents perceived their degree as a disadvantage, these attitudes were measured during the implementation phase and do not fully reflect the post-SAS landscape, which appears more favourable based on recent match and enrolment data [27].

### Pathology

Pathology was largely unaffected by the Single Accreditation System, as the AOA had not accredited any programmes in this specialty for several years before the merger. DO students interested in pathology therefore applied exclusively to ACGME-accredited residencies both before and after SAS implementation. The recent growth in DO representation from 2011 to 2020, more than doubling to 12.1% of all pathology residents, reflects declining interest among US MD graduates rather than structural changes from the SAS (Table 1) [29].

In response to these workforce shortages, many programme directors began filling unoccupied positions with qualified IMGs and DOs. This need also motivated the College of American Pathologists to launch the ‘Pathologist Pipeline Initiative,’ aimed at increasing student exposure, mentorship, and research opportunities. While pathology offers an example of rising DO representation, its trajectory is driven by workforce dynamics rather than the SAS itself [29,30].

## Conclusion

The SAS represented a landmark change in graduate medical education, unifying training standards and expanding application access for DO and MD graduates. While measurable gains have occurred in certain fields, most notably family medicine and pathology, disparities persist in many competitive specialties, particularly those with fewer legacy osteopathic programmes [9,14,22,29,31]. These differences are shaped by multiple factors, including specialty competitiveness, research expectations, and the availability of home programmes, rather than by the SAS alone [15,32,33].

While this review provides a broad synthesis of specialty-specific trends following the SAS, several additional limitations warrant acknowledgement. First, incomplete or absent data for certain specialties, particularly urology, anaesthesiology, and other subspecialties not represented in the current literature, limit the generalisability of our conclusions across the full spectrum of graduate medical education. These omissions reflect true gaps in published research rather than intentional exclusion. Even among the specialties included in this review, the evidence base is not uniformly distributed, which necessarily influenced the depth of analysis available. The literature disproportionately concentrates on surgical specialties, resulting in greater detail within those sections and more limited synthesis for non-surgical fields. Second, portions of the included evidence, such as survey-based assessments of programme director or resident perceptions, are subject to self-selection and response bias. These unmeasured biases may influence reported attitudes toward DO applicants and thus should be interpreted cautiously. Finally, as this is a narrative rather than systematic review, synthesis relied on available published data without quantitative pooling, which constrains the ability to infer causality. Despite these limitations, the review highlights consistent structural and specialty-level patterns that merit continued investigation.

Future research should focus on quantifying the long-term effects of the SAS using specialty-specific metrics beyond match outcomes. These include longitudinal tracking of DO representation in academic faculty positions, programme leadership, and fellowship placement, as well as changes in geographic distribution of the physician workforce. Additionally, there is a need for new primary studies examining how institutional characteristics such as the presence of home residency programmes, research infrastructure, and mentorship accessibility affect residency match equity between DO and MD graduates. Such data would help clarify whether observed disparities are transient byproducts of the merger or persistent structural inequities requiring targeted policy intervention.

Moving forward, addressing disparities will require targeted, evidence-based strategies rather than broad, undefined reforms. Examples include expanding mentorship networks for DO students, building research capacity at osteopathic institutions, and fostering stronger partnerships between osteopathic schools and ACGME-accredited programmes. Continued monitoring of specialty-specific match outcomes, geographic distribution, and leadership representation will be critical to assessing the SAS’s lasting impact on equity and access in graduate medical education.

## Data Availability

There is no data associated with this research.
